# Synergic Effect of TiO_2_ Filler on the Mechanical Properties of Polymer Nanocomposites

**DOI:** 10.3390/polym13122017

**Published:** 2021-06-20

**Authors:** Cristina Cazan, Alexandru Enesca, Luminita Andronic

**Affiliations:** 1 Renewable Energy Systems and Recycling Research Center, Transilvania University of Brasov, 500036 Brasov, Romania; 2 Product Design, Mechatronics and Environment Department, Transilvania University of Brasov, 500036 Brasov, Romania; aenesca@unitbv.ro (A.E.); andronic-luminita@unitbv.ro (L.A.)

**Keywords:** polymer nanocomposites, TiO_2_ nanoparticle, organic–inorganic interfaces, surface modification of TiO_2_ nanoparticles

## Abstract

Nanocomposites with polymer matrix offer excellent opportunities to explore new functionalities beyond those of conventional materials. TiO_2_, as a reinforcement agent in polymeric nanocomposites, is a viable strategy that significantly enhanced their mechanical properties. The size of the filler plays an essential role in determining the mechanical properties of the nanocomposite. A defining feature of polymer nanocomposites is that the small size of the fillers leads to an increase in the interfacial area compared to traditional composites. The interfacial area generates a significant volume fraction of interfacial polymer, with properties different from the bulk polymer even at low loadings of the nanofiller. This review aims to provide specific guidelines on the correlations between the structures of TiO_2_ nanocomposites with polymeric matrix and their mechanical properties. The correlations will be established and explained based on interfaces realized between the polymer matrix and inorganic filler. The paper focuses on the influence of the composition parameters (type of polymeric matrix, TiO_2_ filler with surface modified/unmodified, additives) and technological parameters (processing methods, temperature, time, pressure) on the mechanical strength of TiO_2_ nanocomposites with the polymeric matrix.

## 1. Introduction

Polymer nanocomposites represent a new class of composite materials that generally exhibit better properties than traditional microcomposites, in terms of mechanical properties, thermal and dimensional stability, fire and chemical resistance, optical and electrical properties, etc. Polymer nanocomposites with inorganic fillers attracted significant an attention due to their unique properties and their numerous applications in modern technology. The properties of polymer nanocomposites are mostly a simple combination of incorporated inorganic nanoparticles and polymeric matrix.

Polymeric materials can be used as matrices in nanocomposites due to their good thermal stability, environmental resistance (durability), and electrical, chemical and mechanical properties [1]. However, it is well known that some polymers (e.g., epoxy resin) are highly brittle. This disadvantage limits the application of these polymers in products that require high impact and fracture strength. Inorganic filler added into polymer matrix improved the mechanical performance of the polymeric nanocomposites. Nanofillers have large surface areas, making them chemically active, and making them interact more easily with the matrix [2]. There are many methods to reinforce polymers with rigid fillers to reduce the cost of production, alleviate some of the polymers limitations and expand their applications [3]. How fillers influence the characteristics of these polymers depends on the polymer nature and the proportion of the filler. Fillers are used to modify many properties of polymers, such as mechanical [4] (flexural strength, tensile modulus, tensile strength, fracture toughness and impact energy), thermal, electrical, and magnetic properties [5,6].

The polymer mass, chemical structure, semi-crystallinity, chemical solubility, and thermal stability, and the nanoparticle surface area, chemical structure, and dispersion are essential for obtaining polymer nanocomposites and understanding their behavior. There are several methods to obtain polymeric nanocomposites, such as modified emulsion polymerization [7], in situ polymerizations [8,9], via direct blending (mechanical mixing) [10,11], solution dispersion [12,13,14], the sol-gel method and melt compounding [15,16], selective laser sintering process [17], and melt extrusion and injection molding [18,19]. Each process is specific, but the final morphology of the nanocomposites plays an important role. The morphology depends not only on the method of obtaining the nanocomposites, but also on the polymer–nanoparticle interactions that promote good dispersion and distribution of the nanoparticles in the polymer matrix [20,21].

Polymer nanocomposites have superior mechanical and physical properties over host polymers, due to the large interfacial area between the polymer matrix and nano-fillers.

Among the different fillers used, such as clays, silicas, nanotubes, inorganics, etc., titanium dioxide (TiO_2_) play a special role in polymeric matrices, to synthesize high-performance and malleable polymer networks (e.g., improving viscosity, obtaining filaments for 3D printing) [22,23,24]. TiO_2_ is found in many applications due to its good photocatalytic properties, hence it is used in antiseptic and antibacterial compositions, degrading organic contaminants and germs, as a UV-resistant material; this is due to its chemical inertness properties, non-toxicity, low cost, high refractive index, and other advantageous surface properties. In these applications, TiO_2_ is used as a component of various types of nanocomposite materials with special properties, which open up opportunities in the following various fields of applicability: in the production of pharmaceuticals, cosmetics or paints [25], drug delivery systems with controlled release [26], solar cell [27], chemical sensing, luminescent material, and photocatalyst [28]. For example, as materials for obtaining membranes for integration in environmental applications, including water treatment or reducing humidification [29,30]. Polymer nanocomposites find applications in the development of optical and electronic devices, sensors, and bio-sensors [31,32].

The incorporation of TiO_2_ nanoparticles into different types of the polymeric matrix could produce synergistic effects. Studies have been performed on the TiO_2_ nanoparticle effect on several properties of polymeric composite, mainly to figure out whether the application of nanoparticles can enhance the mechanical performance of polymeric composites for applications in various fields.

This paper comprehensively reviews some essential aspects, such as the processing, characterization, and mechanical properties of various nanocomposites with a polymeric matrix and TiO_2_ fillers.

## 2. Polymeric Matrix

### 2.1. Matrix

The main component in the nanocomposite of the polymer matrix is the polymer itself. There are many varieties of polymers used in the preparation of polymeric matrix nanocomposites. These polymers can be thermoplastics, thermosetting, elastomers, natural, and biodegradable polymers. The choice of filler depends on the nature of the polymer, thus obtaining materials with the following specific properties: mechanical, electrical, magnetic, optical biocompatibility, chemical stability, and functionalization. Thermosetting polymer nanocomposites are usually the most common nanocomposites. They are used in many applications, but recently thermoplastic polymer nanocomposites have attracted much of the research interest in industry and academia. The properties of polymers depend mainly on the polymer structure, which in turn depends on the chemical composition, surface morphology, and processing parameters. Polymers are a source of a wide variety of low-priced raw materials, which offer many advantages, such as the following [10]: low specific weight, high material stability against corrosion, good electrical and thermal insulation, ease of shaping and economical mass production, and attractive optical properties. However, they have some deficiencies in strength and stiffness. Fillers are integrated into polymer materials to make up for those deficiencies. These polymers can be epoxy resins, polyester fibreglass resins systems, PURs, PIs, urea, etc.

Theoretically, the associations that can be made between different polymers and the wide range of fillers are infinite. In practice, however, although numerous, the polymer–filler associations are limited. Among the thermoplastic polymers for the processing of which fillers can be introduced, the most important are as follows: polyolefin, polyamides, ABS polymers, polyesters, polycarbonates, and PVC. Elastomers are flexible polymers that comprise a low crosslink density and generally have low Young’s modulus, and by incorporating the fillers, these matrices can be more resistant [11].

### 2.2. Matrix–Filler Interface

**Figure 1:**
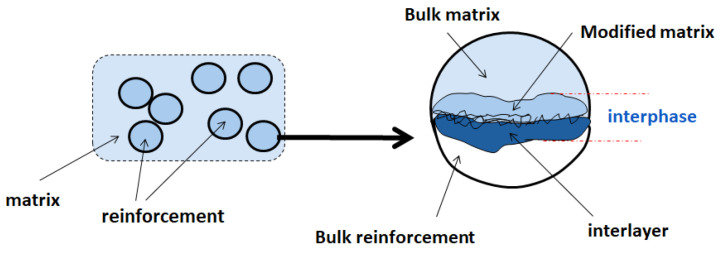
Representation of the interphase between matrix and fillers.

The nature of the interface between the matrix and filler is an essential factor influencing the nanocomposite properties. According to Sharpe [33], the interface is defined as an intermediate region of two phases in contact, whose composition, structure, and properties vary throughout the area and are generally different from the two phases. Such phases are rarely devoid of chemical interaction. The volume of material affected by the interface interaction forms a a three-dimensional zone, called the interphase. The term interphase is widely used in the adhesion community to indicate the presence of a chemically or mechanically altered zone between adjacent phases [34,35]. The interphase concept, according to Drzal [36], is schematically represented in Figure 1. 

Knowledge of the relationship between microstructure and properties in the interface region is essential for the correct use of composite materials. There is no simple quantitative relationship for interface optimization that combines polymeric matrix and fillers [16]; the physicochemical variation and the thermodynamic–mechanical principles are sources of information for the qualitative assessment of the interface phenomena. Numerous researches have been carried out to improve the properties of the composites, particularly the interface when the filling is inorganic, for example, TiO_2_ [17].

**Figure 2:**
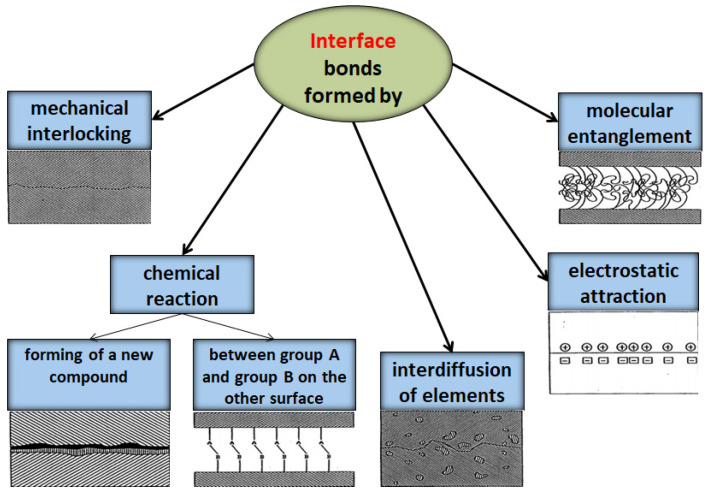
The formation of the interface between matrix and filler.

Studies have shown [37,38] that the interface has different properties from both the matrix and the filler material. This consists of several layers that can each affect the adhesion of the components. The filler–matrix bonding depends on the following physicochemical aspects of the interfaces of the composite: atomic arrangement, molecular conformation, the chemical constitution of the fillers, matrix and fillers morphological properties, and the diffusivity of the elements in each constituent. The adhesion between the polymeric matrix and the dispersed phase particles was explained in mechanical and thermodynamic adhesion, chemical compatibility, chemical reactions with new bonds, electrostatic attraction forces, and macromolecular interdiffusion, adsorption and watering, as shown in Figure 2 [39]. The mechanical coupling or interlocking adhesion mechanism is based on the adhesive keying into the surface of the substrate [40] and locking the rough irregularities on the surface of the nanocomposites. In many studies, it was shown that the adhesion mechanism was due to interchain entanglement and not chemical bonding between the components of the composites. The mechanical adhesion primarily depends on the forces in the transition region between the non-contacting areas [41,42]. The thermodynamic mechanism assumes that it does not require a molecular interaction for good adhesion, only an equilibrium process at the interface [43]. In neutral environments, such as air, the thermodynamics of the polymer system will attempt to minimize the surface free energy by orientating the surface into the non-polar region of the polymer. When the polymer surface is in contact with a polar substance, such as water, good adhesion requires that the interfacial tension be minimized [44]. The other theories mentioned above are explained based on the physico–chemical interactions between the components of the composites.

### 2.3. Fillers and Surface Modifications

Composite materials with optimal performances are obtained if an optimal adhesion between the matrix and the filler is achieved. Optimal adhesion is realized between materials of close chemical nature. It is weak between very different materials, from a chemical point of view, such as between the polymer matrix and inorganic fillers. Thus, the adhesion can be improved by treating the surface of the dispersed particles with coupling agents. Surface modification of fillers is becoming more important because of its adhesion improvement on the stress transfer between polymer and filler, which leads to an increase in the dispersion degree [45]. The coupling agent diffusion and adsorption processes at the surface of the filler particles occur at the interface. The properties of the interface and the adhesion of the components can be modified by treating the surface of the fillers before introduction into the polymer matrix. These treatments either remove the weaker layers related to the filler surface of the material or introduce new functional groups capable of influencing the adhesion between the materials.

Surface treatment of the fillers can be achieved by [46,47] the following:

− The chemical interaction of the fillers with compounds that possess functional groups;
− Chemical absorption on the surface of the particles of the filling material of some modifying agents;
− Coating the filler particles with a suitable coupling agent.

These processes are generally laborious and increase the cost of the fillers, but they offer the possibility of considerably increasing the fillers content in mixtures without worsening their characteristics.

Modification of the surface of fillers is becoming more important because of its improvement in adhesion [48]. Hence, it is on the stress transfer between the polymer and filler, leading to an increase in the dispersion degree.

## 3. Titanium Dioxide Nanoparticles

### 3.1. Size, Shape and Specific Surface Area of the Nanoparticles

Titanium dioxide (TiO_2_) is the natural oxide of the element titanium. Titanium dioxide adopts four structures polymorphs found in nature rutile, anatase, brookite, and TiO_2_ (B). An additional four high-pressure forms have been synthesized, as follows: TiO_2_ (II) with the α-PbO_2_ structure, TiO_2_ (H) with hollandite, baddeleyite with ZrO_2_, and cotunnite with PdCl_2_ [49]. Among the eight structures, rutile and anatase are mostly manufactured in the chemical industry as microcrystalline materials. Thermodynamically, rutile is the most stable phase at all temperatures and pressures below 60 kbar, when TiO_2_ (II) becomes the favourable phase. Particle size influences surface energy and phase stability. Thus, anatase is most stable at sizes less than 11 nm, brookite at sizes between 11 and 35 nm, and rutile at sizes greater than 35 nm. Anatase and brookite are more stable than rutile at nano-size, due to the differences in surface energy. Anatase is more stable than brookite at even smaller sizes [50]. From a commercial point of view, titanium dioxide can be found in the following two common forms that differ in crystal structure: anatase and rutile [51,52,53].

Titanium dioxide can be prepared in the following various morphologies: nanoparticles, nanowires, nanotubes, and mesoporous structures. There are physical and chemical methods for synthesizing TiO_2_ nanoparticles in the liquid phase, as follows: hydrothermal/solvothermal method, sonochemical method, electrochemical synthesis, sol-gel method, microwave field synthesis, and vapor phase, which includes spraying, atomic deposition of layers, pulsed laser deposition, chemical vapor deposition, physical vapor deposition, and pyrolysis spray [54,55]. The controllable synthesis of TiO_2_ with unusual morphologies and dimensions can give the polymeric matrices with particular features and qualities.

The specific surface area of TiO_2_ increases as the particle size decreases, meaning nanoparticles are attracted due to van der Waal electrostatic forces. With the decreasing particle size, the ratio of surface/volume increases. Therefore, the smaller the particles are, the more important the surface properties will be, influencing agglomeration behavior and interfacial properties as a result of interaction with the polymer matrix [56,57]. The formation of particle agglomerates and non-uniform dispersion has motivated research to better process polymer–TiO_2_ nanocomposites. Several methods have been approached to minimize agglomeration and ensure better distribution. Such methods may be as follows: melt mixing, solution mixing in aqueous media or polymer matrices, particle surface modification involving polymer surfactant molecules or other modifiers, which must generate a strong repulsion between nanoparticles, mechanical stirring, and ultrasonic irradiation.

### 3.2. Surface Modification of TiO_2_ Nanoparticles

TiO_2_ nanoparticles can be directly added to the organic matrix, but due to the high surface area and high polarity, there is a strong tendency for them to aggregate. TiO_2_ nanoparticles form agglomerates at higher concentrations due to their high surface energy. Surface modification of TiO_2_ nanoparticles effectively reduces their surface energy and improves their dispersion properties in the organic matrix. Therefore, to improve the homogeneous dispersion of nanoparticles, many researchers have focused on the surface modification of nanoparticles and a new method for incorporating inorganic nanofiller into an organic matrix [58,59,60]. Several ways have been employed to modify the surface of nanoparticles [61,62].

The surface modification of TiO_2_ nanoparticles is often conducted by either a physical or chemical method. The chemical method has attracted the attention of many researchers because the interactions between inorganic nanoparticles and the matrix are much stronger [63]. The surface modification of nanoparticles by chemical treatments is a useful method to improve the dispersion stability of TiO_2_ nanoparticles and the development of interfaces between the organic and inorganic phase. In this regard, the concept of silane coupling agent was reported by Plueddemann and et al. [64]. Researchers found that organofunctional silanes are silicon chemicals that contain both organic and inorganic reactivity in the same molecule, and which can be used as coupling agents [65,66]. Coupling agents connect resin and fillers, and improve the physical, mechanical and electrical properties of composites. Moreover, they enhance the wetting of inorganic substrates, decrease the viscosity of the resin during mixing, and ensure smoother surfaces of composites [67,68].

**Figure 3:**
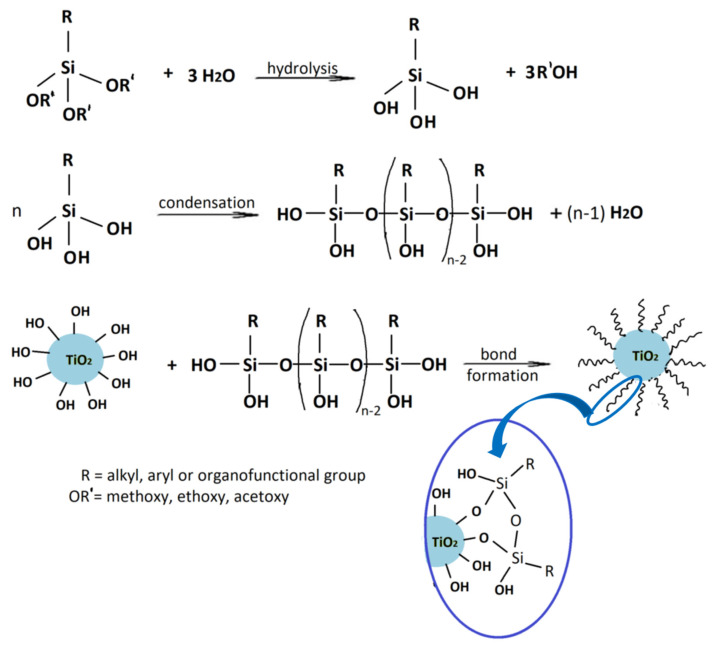
The interaction between the coupling agent molecule and the filler.

The general formulation of the coupling agent molecule is as X–R, where X interacts with the filler and R is compatible with the polymer. Organosilanes are of the form R–Si–(OR’)_3_, where OR’ can be methoxy, ethoxy, acetoxy, and R can be alkyl, aryl or organofunctional group [56]. According to this structure, the following steps may take place, as shown in Figure 3:

✓ Hydrolyzation of alkoxy groups obtaining silanol, which reacts with the mineral surface;
✓ The condensation reaction between silanol molecules;
✓ Bond formation between TiO_2_ nanoparticles and the organofunctional group.

The choice of organosilane is established, taking into account the polymers chemical structure to be compatible. For example: for a phenolic and epoxy resin an epoxy silane, or an amino silane is recommended and for an unsaturated polyester resin a methacrylsilane. The reactivity of the thermosetting polymers should be close to that of organosilane. For a thermoplastic matrix, bonding occurs by diffusion of the organosilane network in the interphase region of the composite [66].

There were silane coupling agents used, such as 3-methacryloxypropyl-trimethoxysilane (MPS) [68], 3-aminopropyltriethoxysiane (APTES) [69], γ-glycidoxypropyltrimethoxysilane (GPS) [70], n-propyltriethoxysilane and 3-methacryloxypropyltrimethoxysilane [71], which change the hydrophilic particles into a hydrophobic surface by providing some molecules with certain hydrophobicity.

Some coupling agent recommendations for the surface modification of TiO_2_ nanoparticles is given in Table 1. 

Silane coupling agents are usually employed to realize chemical modification. These can offer hydrolyzable groups bonding with the inorganic particles. After bond formation, the organosilane functional groups of silane coupling agents form a hydrophobic layer on the surface of the inorganic nanoparticles. Different coupling agents have been used to modify the surface of TiO_2_ and improve the interfacial interactions necessary for the successful incorporation of these hydrophilic nanoparticles into hydrophobic polymer matrices.

The surface modification of TiO_2_ has been reported using different silane coupling agents, such as 3-aminopropyltriethoxysilane (APTES). The photocatalytic activity of TiO_2_ has been shown to increase with increasing the concentration of APTES used [87]. For example, Mallakpour and Barati [88] reported the surface modification of TiO_2_ nanoparticles by the reaction with APTES. The silane coupling agent was adsorbed on the surface of the nanoparticles at its hydrophilic end and interacted with the hydroxyl groups pre-existing on the surface of the nanoparticles. Thus, it was confirmed that the heat stability of the nanocomposite was improved. Shakeri et al. [89] studied the self-cleaning capability of surfaces covered TiO_2_ nanoparticles, modified by APTES. They concluded that the surface could degrade the dye used as an organic pollutant due to the obtained coating being stable. Klaysri et al. [90] proposed a one-step synthesis method of APTES-functionalized TiO_2_ surface. They showed that obtained nanomaterials are capable of the photocatalytic decolonization of methylene blue. 

Modification of the surface of TiO_2_ nanoparticles with silane coupling agents was obtained via reflux in an aqueous solution [75,91]. Chen et al. investigated the interactions between 3-aminopropyltrimethoxysilane (APTMS) and phenyltrimethoxysilane with commercially available TiO_2_ nanoparticles (Degussa P-25) [91]. They obtained results showing that the silane coupling agents used bind covalently on the surface of the TiO_2_ nanoparticles. In another study, Zhao et al. reported the cross-linking and chemical bonding mechanisms of APTMS and 3-isocyanatopropyltrimethoxysilane on TiO_2_ nanoparticles [75]. 

To improve TiO_2_ nanoparticles dispersion and enhance the interactions between the nanoparticles and polymeric matrix (polyamide/modified–TiO_2_ nanocomposites), the surface of TiO_2_ was modified with a 1,3,5-triazine based silane coupling agent [92].

Caris et al. [93] used conventional emulsion polymerization to encapsulate TiO_2_ in poly(methyl methacrylate) (PMMA). Sidorenko et al. [84] investigated the radical polymerization of styrene and methyl methacrylate (MMA). This reaction was initiated at the surface of TiO_2_ particles by adsorbed hydroperoxide macroinitiators. Erdem et al. [94] encapsulated the TiO_2_ nanoparticles by miniemulsion polymerization of styrene, polybutene-succinimide pentamine being used as the stabilizer at the oil/water interface. Rong et al. [95] used the TiO_2_ nanoparticles modified by 3-(trimethoxysilyl) propylmethacrylate (MPS) to copolymerize styrene with the methacrylate group of MPS, by free-radical polymerization. Yang and Dan [96] used a similar approach by graft polymerized MMA on the modified surface of the TiO_2_ nanoparticles. 

Milanesi et al. used a mixture of isomeric octyltriethoxysilanes (OTES), highlighting the hydrophobic layer structure. They concluded that the cross-linking (via Si–O–Si bonds) and chemical bonding (via Ti–O–Si bonds) of silanes onto TiO_2_ nanoparticles occurred [97]. Xiang et al. used 3-methacryloxypropyl-trimethoxysilane (MPS) to modify the TiO_2_ surface to enhance the compatibility of TiO_2_ nanoparticles in the poly(butyl acrylate) (PBA) matrix. The modified TiO_2_ presented good compatibility in the PBA matrix [98]. In another study [83], Xiang showed the hydrophobic surface modification of TiO_2_ to produce acrylonitrile-styrene-acrylate (ASA) terpolymer–TiO_2_ composites for cool materials. Wang et al. [99] functionalized the commercial TiO_2_ nanoparticles in an aqueous solution via ultrasonic treatment at room temperature with 3-(trimethoxysilyl)propyl methacrylate. 

Godnjavec et al. have coated TiO_2_ nanoparticles by 3-glycidyloxypropyltrimethoxysilane (GLYMO) as an additive in a clear polyacrylic coating. According to their results, grafting GLYMO on the nanoparticles surface improved the dispersion, transparency, and UV protection of the clear acrylic coating [100].

Yang et al. [101] reported silanization of TiO_2_ particles through a sol-gel method. Based on their results, vinyl triethoxysilane (VTES) as a surface modifier improved the stability of dispersion and suspension in tetrachloroethylene. Dalod et al. [50] modified TiO_2_ nanoparticles with amino silane groups using a hydrothermal method and found that the nanoparticles shape and structure depends on the type of silane coupling groups. 

Tangchantra et al. [102] investigated the effect of different silane coupling agents on the surface grafting of TiO_2_ with hexadecyl trimethoxysilane (HTMS), triethoxyvinylsilane (TEVS), and aminopropyl trimethoxysilane (APS). The results showed that silane coupling agents could modify the surface of TiO_2_ nanoparticles via the hydrolytic condensation of titanium isopropoxide. The TEVS agent improved the dispersibility of TiO_2_ particles and showed optimum mechanical properties.

The appropriate surface modification on nanoparticles leads to better dispersion and compatibility in the polymer matrix. The formation of chemical and physical interactions with the polymer matrix could guarantee remarkable mechanical properties of polymeric nanocomposites.

### 3.3. Properties, Commercial Products and Applications

At the nanoscale size, the material properties may dramatically change and differ significantly from their bulk counterparts. 

Particular attention has been paid, in recent years, to obtaining TiO_2_ with photocatalytic properties [103,104,105,106], optical properties [107], with applications related to the degradation of pollutants [108,109,110], and the realization of the photoelectrochemical cells [111]. Also of interest are titanium dioxide films deposited on various substrates to obtain special characteristics, such as surfaces with self-cleaning properties [112,113].

All applications of TiO_2_ nanoparticles depend on their crystal structure, morphology, specific surface area, particle size, and form. TiO_2_ has been widely used in the industry for many years for its numerous and diverse applications, as shown in Table 2.

The applications that can be mentioned are sensors, photo-conductors, additives in plastics, catalysts, photo-/electrochromics and photovoltaics applications, dye-sensitized solar cells, sunscreens, paints, antimicrobial applications, water purification by photocatalysis processes, biosensing, and drug delivery [114]. TiO_2_ nanoparticles incorporated into outdoor building materials, such as paving stones or paints, can reduce volatile organic compounds and nitrogen oxide concentrations.

TiO_2_ is a material with multifunctional properties that can be incorporated in polymeric matrices as a filler to develop new nanocomposites with enhanced properties [115].

## 4. Polymeric Nanocomposites with TiO_2_ Filler

### 4.1. Preparation Methods

Polymeric matrix nanocomposites can be obtained using injection molding, compression molding, in situ polymerization, sol-gel, melt mixing and sintering. 

In situ polymerization involves the dispersion of inorganic nanoparticles in a monomer phase as a first step, followed by bulk phase polymerization. This process is mainly used for thermosetting polymers. As a result, unstable nanocomposites can be transform into a different morphology than expected. The in situ polymerization method is a simple and inexpensive method. The nanocomposites with the polymer matrix, and inorganic filler with good filler distribution in the polymer matrix, can be obtained [116].

Most compression molding techniques require pre-treatment of the nanoparticles with curing, but injection molding is the most widely used process for obtaining nanocomposite materials. Injection molding can be used in a variety of applications, in both commercial and research fields [117]. Sintering, powder compaction and sol-gel are all alternative techniques to produce polymeric composites. However, the operating conditions (temperature, pressure, time, etc.) are far more than those of injection molding [118]. Some reports were found in the literature focusing on obtaining TiO_2_ nanocomposites with the polymeric matrix, as shown in Table 3.

Studies on polymer–TiO_2_ nanocomposites prepared by melt mixing have shown a slight improvement or no change in mechanical properties [119,120]. Somani et al. [121] received highly piezoresistive conducting polyaniline/TiO_2_ composite by in situ deposition technique at a low temperature. Feng et al. [122] synthesized a composite of polyaniline-encapsulating TiO_2_ nanoparticles by in situ emulsion polymerization. They investigated and explained the interaction between polyaniline and nano-TiO_2_ particles, and the nature of chain growth according to Fourier transform infrared (FTIR) spectra. Xia and Wang [123] prepared a polyaniline/nanocrystalline TiO_2_ composite by ultrasonic irradiation. They think that ultrasonic irradiation provides a new way to prepare 0–3-dimensional conducting polymer/nanocrystalline composites.

Titanium dioxide has been used to reinforce polypropylene (PP) via extrusion, followed by injection molding, by Alghamdi [124]. There were presented to the mechanical and structural aspects of PP for different loading of TiO_2_ filler (up to 30 wt. %). As the TiO_2_ weight percent increases, the impact strength decreases. This behaviour is expected because the PP is incompatible with TiO_2._ The PP phase is non-polar, hydrophobic and has low surface energy, while TiO_2_ represent the polar phase, hydrophilic and high surface energy for TiO_2_. The highest resilience value was recorded for the sample with 20% TiO_2_ (37.09 ± 5.3 J/m). 

Mourad et al. [117] studied HDPE nanocomposites with 5% TiO_2_, obtained by injection molding under the following different processing parameters: temperature, pressure, injection velocity, and injection time. The results showed the influence of processing parameters on the mechanical and thermal properties of HDPE–TiO_2_ nanocomposites. Mechanical testing revealed that the tensile strength varied from 22.5 to 26.3 MPa, while the Young modulus increased by 8.6% as the molding temperature increased. 

Vladuta et al. [125] investigated the effect of the TiO_2_ nanoparticles on the PET–rubber interface in nanocomposites obtained from waste by compression molding. The modifications in surface energy, morphology and crystalline structure were discussed for samples kept under visible light and UV radiation. TiO_2_ develops new physical interactions in the composite, but induces, even in visible light, oxidation processes. The results indicated that the optimum concentration for TiO_2_ to the composites, for obtaining better interface properties, is 0.25 wt. %.

Regardless of the method of obtaining nanocomposites with the polymeric matrix, it is found that the nature of filler has a significant influence on mechanical properties.

### 4.2. Mechanical Properties

**Figure 4:**
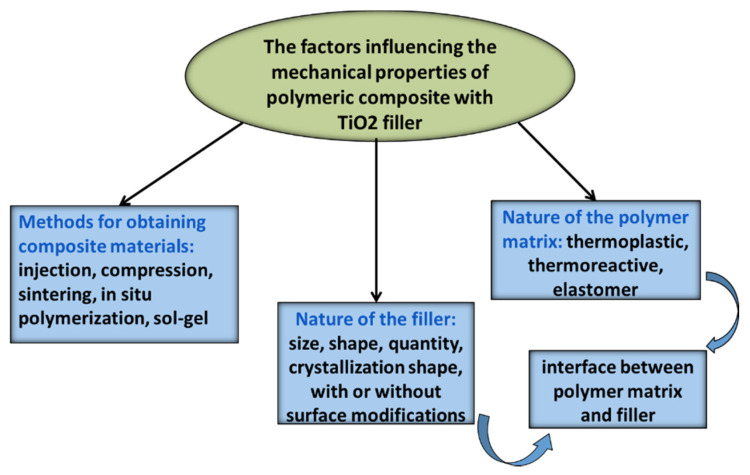
The factors that influence the mechanical properties of composites materials.

Titanium dioxide is used as a filler in many polymeric matrices because of the improved physical and mechanical properties it yields. Many studies showed improvements in the mechanical strength and modulus of TiO_2_-filled polymeric nanocomposites compared to the pristine-base matrix. The mechanical properties of the TiO_2_ nanocomposites depend significantly on their internal structure. The poor compatibility of hydrophilic TiO_2_ nanoparticles with a hydrophobic polymer matrix may lead to particle aggregates and/or agglomerates. The aggregates create defect sites in the nanocomposites, and the improvement in mechanical properties is not observed. More uniform dispersion of nanoparticles is recommended, using one-dimensional nanoparticles, i.e., nanorods, nanotubes or nanoribbons, particles with a high aspect ratio [46]. Several factors that may influence the mechanical properties of composites with a polymer matrix and inorganic fillers are presented in Figure 4.

#### 4.2.1. The Nature of the Filler

TiO_2_ fillers affect the basic mechanical properties of the polymer. The effect of TiO_2_ fillers on composites properties depends on the particle size and shape, concentration and the interaction with the matrix, as shown in Table 3. For example, to increase the modulus and hardness of polymers, micrometre-sized inorganic particles are frequently applied. However, a reduction in the material ductility may take place. By diminishing the particle size or by enhancing the particle volume fraction, the strength can be improved. Still, in some cases, the fracture toughness and modulus remain relatively independent of the particle size. The properties of TiO_2_ that make it a good filler for composite materials are good dispersibility in the polymer system and good heat stability. Titanium dioxide has a relatively high elastic modulus, which can be frequently combined into various polymers to obtain the composites mechanical gain.

Mikešová and et al. [126] studied the effects of nanoparticles and the properties of the nanocomposites of polypropylene and filler TiO_2_. They used isotactic polypropylene (PP) as a matrix, and as fillers they used TiO_2_ in the following different shapes: a commercial titanium dioxide micropowder (mTiO_2_; a mixture of anatase and rutile), a commercial titanium dioxide nanopowder (nTiO_2_; anatase modification), and titanate nanotubes (TiNT). More series of samples were obtained with PP unmodified and with PP modified by electron beam irradiation (PP*), resulting in PP*/TiX composites (i.e., PP*/mTiO_2_, PP*/nTiO_2_, and PP*/TiNT). These were prepared by melt mixing of PP* with 5 wt. % of TiX. The stiffness and microhardness properties of PP*/TiX systems are improved in the order PP*/mTiO_2_, PP*/nTiO_2,_ PP*/TiNT, due to the specific surface of the TiX particles.

Nano-sized TiO_2_ was further studied in starch/(poly[vinyl alcohol]) blends by Sreekumar et al. [127]. The nano-sized TiO_2_ could provide the composite with superior mechanical properties because of good interfacial adhesion between the polymer matrix and filler.

Bora et al. [128] studied the effect of TiO_2_ particle concentrations (up to 25 wt. %) on the properties of polyphenylenesulphide (PPS)–TiO_2_ composites. The increase in TiO_2_ particle concentrations in the PPS matrix improves the stiffness of the composite. High values of flexural and residual flexural strength were obtained at 10 wt. % TiO_2_ particle concentrations. Saluja et al. [129] obtained polyester composites filled with TiO_2_ concentrations up to 25 wt. %. This study shows that the addition of TiO_2_ particles improves the effective thermal conductivity of polyester–TiO_2_ composites, the glass transition temperature (Tg), and the reduction in the coefficient of thermal expansion (CTE).

The mechanical properties of nanocomposites depend significantly on their internal structure. In the nanocomposite, TiO_2_ nanoparticles can appear as agglomerations due to their low compatibility with the hydrophobic polymer matrix.

In this case, the large surface area of the nanowires decreases rapidly, the aggregates create defect sites in the nanocomposites, and no improvement in the mechanical *properties* is observed. A more uniform dispersion of nanoparticles, using one-dimensional (1D) nanoparticles, i.e., nanorods, nanotubes, or nanoribbons, would improve these properties. Compared with the isometric nanoparticles, a large surface-to-volume ratio of the 1D nanoparticle generally improves the nanocomposites properties. Contrary to the anatase polymer nanocomposites, only a few papers concerning polymers filled with titanate nanotubes have been found in the literature [130,131,132].

The majority of nanoparticle fillers added in the polymer matrix improve mechanical properties such as flexibility, ductility, hardness, and strength and stiffness, even in small amounts.

#### 4.2.2. The Nature of the Polymer Matrix

Polymer–TiO_2_ nanocomposites have been successfully synthesized in different polymer matrices such as the following thermoplastic polymers: polyacrylate, poly (methyl methacrylate), polyimide, polystyrene, and polyolefines; the following thermosetting polymers: polycarbonate, polyamide 6, epoxy, unsaturated polyester; and silicone elastomer [77,132].

Saritha et al. [133] studied the incorporation of TiO_2_ in rubber composites. The tensile strength, modulus, and tear strength increased with increasing TiO_2_ loading. More recently, processing techniques were developed to allow the size of TiO_2_ to decrease to the nanoscale. Manap et al. [134] demonstrated that TiO_2_ and multi-walled carbon nanotubes (MWCNT) as filler reinforcements could address the agglomeration issue, by exhibiting even distribution of particles in the TPU matrix. The combination of MWCNT and TiO_2_ in the TPU matrix enhanced the mechanical and thermal properties significantly, this being a good heat insulator.

In the function of the matrix nature, the percentage by weight of the inorganic filler introduced can remain very low (on the order of 0.5% to 5%) due to the incredibly high surface area-to-volume ratio of the particles. This area can generate a new material behavior, which is widely determined by interfacial interactions, offering unique properties and an entirely new class of materials. Several important types of research in this regard are presented in Table 3.

**Table 3:** Types of nanocomposites with polymeric matrix and TiO_2_ filler.

Composites	Materials	Methods	Results-Mechanical Properties	Ref.
Thermoplastic matrix				
Polystyrene (PS)–TiO_2_	*Matrix*: polystyrene. *Filler:* TiO_2_ (0.19 µm) *Coupling agent:* 3-amino ethoxy silane (0.1, 0.5 and 1 wt. %.).	*Obtaining:* mixing of matrix with TiO_2_-coupling agent *Characterization:* mechanical tests, SEM analysis.	Values of Young’s modulus, tensile strength, elongation at break, flexural strength increase with linearly filler concentration followed by a decrease beyond 15 wt. %.	[45]
Polyphenylene sulfide (PPS)–TiO_2_	*Matrix:* polyphenylenesulphide (PPS) *Filler:* TiO_2_ (200 nm; 0, 5, 10, 15, 20, and 25 wt. %)	*Obtaining:* injection molding. *Characterization:* solid particle erosion test, three-point bending test, thermal analyzing methods.	The flexural modulus of composites increased with the increase in TiO_2_ concentration up to 10 wt. %, and then it decreases. TiO_2_ filler caused to reduce the erosion resistance of the PPS composites.	[128]
Polypropylene (PP)–TiO_2_	*Matrix:* PP pellets; *Filler:* TiO_2_ (0, 10, 20 and 30 wt. %)	*Obtaining:* injection molding *Characterization:* mechanical properties: tensile stress, impact tests; TGA	The highest resilience value recorded for the sample with 20% TiO_2_ (37.09 ± 5.3 J/m). Tensile stress shows a decrease and the E modulus increase as the weight percent of TiO_2_ increases.	[124]
Polypropylene (PP)–TiO_2_	*Matrix:* polypropylene (PP) *Filler:* TiO_2_ micropowder; TiO_2_ nanopowder titanate nanotubes (TiNT)	*Obtaining:* melt mixing; samples types PP*/TiX (PP*/mTiO_2_, PP*/nTiO_2_, PP*/TiNT) and samples with PP unmodified. *Characterization:* SEM analysis, TEM analysis, mechanical properties	The stiffness and microhardness of the PP–TiNT nanocomposites increase by 27% and, respectively, 33%. In the PP–nTiO_2_ nanocomposites, the increase in these mechanical characteristics is lower.	[126]
Polypropylene (PP)–TiO_2_	*Matrix:* PP homopolymer *Filler:* TiO_2_ (0–3 wt. %)	*Obtaining:* melt compounding; *Characterization:* mechanical properties, thermogravimetric analysis, DSC, SEM analysis	The addition of TiO_2_ nanoparticles increases the mechanical properties of PP fibres. Tenacity is increased by 72.69% for the PP–TiO_2_ (3 wt. %) nanoparticle. Elongation at break of the PP fibres with TiO_2_ (1.5 wt. %) indicated an increase of 15.79%.	[135]
Polypropylene (PP)-rice husk–TiO_2_	*Matrix:* polypropylene (PP) *Filler:* rice husk and TiO_2_	*Obtaining:* injection molding *Characterization:* mechanical properties, SEM, TGA	Incorporating inorganic filler TiO_2_ into PP/RH significantly enhanced the green hybrid PP/RH/TiO_2_ composites mechanical properties and thermal stabilities. The maximum values of tensile strength and Young modulus were 41.2 MPa for PP/RH (10wt. %)/TiO_2_ (3wt. %), respectively, for PP/RH (40wt. %)/TiO_2_ (3wt. %)	[136]
polyurethane (TPU)–TiO_2_	*Matrix:* polyurethane (TPU) matrix with multi-walled carbon nanotube (MWCNT); *Filler:* TiO_2_ (particle diameter—0.19 μm).	*Obtaining:* injection molding. *Characterization:* mechanical properties: tensile test, DMA, TGA,	The composites have good mechanical properties: tensile stress was 4.46 MPa, elongation at the break—49%, and Young’s Modulus— 9.17 MPa.	[134]
thermoplastic polyurethane (TPU)–TiO_2_	*Matrix:* thermoplastic polyurethane *Filler:* TiO_2_ nano-particles *Coupling agent:* aminopropyl trimethoxy silane (APS)	*Obtaining:* mixing of matrix with filler; *Characterization:* elemental analysis, FTIR spectroscopy, TGA, mechanical properties.	For composite with TiO_2_ (3 wt.%), tensile strength and Young’s modulus were increased by 72% and 48.9, respectively. Higher values were obtained when modified TiO_2_ was used, at low percentages (1 wt.%).	[77]
polybutylene succinate (PBS)–TiO_2_	*Matrix:* polybutylene succinate (PBS); *Filler:* TiO_2_ (20 nm; 0, 0.5, 1, 2, 5, and 10 wt. %)	*Obtaining:* vane extruder. *Characterization:* SEM, TEM, XRD, DSC, TGA, DMA; mechanical test, UV transmittance.	TiO_2_ has little effect on the impact strength of the composite material. The flexural modulus of composites improved by 36.3% with TiO_2_ (10 wt. %) addition. The tensile modulus of PBS–TiO_2_ (10 wt. %) was higher by 15.5% than that of pristine PBS.	[137]
polyetheretherketone (PEEK)–TiO_2_	*Matrix:* PEEK. *Filler:* TiO_2_ powder (1, 3, 5 wt.%)	*Obtaining:* mixing and extrusion forming; *Characterization:* density and Melt Flow Index (MFI) measurement, DSC, UV thermal, mechanical test	E modulus increase with TiO_2_ content. The PEEK-1% TiO_2_ sample has a tensile strength higher than that of pristine PEEK. TiO_2_ (5% vol.) particles act effectively as UV blocker retarding the photo-degradation of PEEK.	[138]
poly(ethylene terephthalate) (PET)–TiO_2_ Poly(lactic acid) (PLA)–TiO_2_	*Matrix:* poly(ethylene terephthalate) (PET) and poly(lactic acid) (PLA); *Filler:* TiO_2_ (20 nm);	*Obtaining:* extrusion forming; *Characterization:* analysis—DSC, XRD, SEM, DMTA, UV–Visible test, mechanical test.	The mechanical properties of PET–TiO_2_ and PLA–TiO_2_ composites have maximum values at a loading level of 3% TiO_2_.	[139]
poly(L-lactide-co-ε-caprolactone) (PLCL)–TiO_2_ nanocomposites	*Matrix:* PLCL; *Filler:* TiO_2_ (20 nm) *Coupling agent*: silane coupling agent NH_2_(CH_2_)_3_Si(OC_2_H_5_)_3_	*Obtaining:* solution casting method. *Characterization:* analysis—FTIR, DSC, TEM, tensile test, shape memory;	For composite with TiO_2_ (5%) the ultimate tensile strength and the elongation at break increase to 35.4 MPa and 444.6%, which are 113% and 11% higher than that of pure PLCL.	[140]
Poly(L-Lactide) (PLLA)–TiO_2_	*Matrix:* poly(L-Lactide) (PLLA) *Filler:* TiO_2_ (<25 nm particle size) and Halloysite nanoclay (HNT) (Al_2_Si_2_O_5_(OH)_4_.2H_2_O);	*Obtaining:* compression molding. *Characterization:* mechanical test	Young modulus had a significant increase (*p* ≤ 0.05) with the addition of TiO_2_ up to 2.5 g TiO_2_/100 g PLLA. Regarding the tensile strength, better results were also achieved when adding 2.5 g TiO_2_/100g PLLA.	[141]
Poly(lactic acid) (PLA)–TiO_2_	*Matrix:* PLA (4032D, 1.2–1.6% D-isomer lactide) *Filler:* TiO_2_ (20 nm);	*Obtaining:* injection molding; *Characterization:* SEM, TEM, dynamic rheological measurements, DSC, TGA, tensile testing, UV transmittance	Samples show a higher elongation at break, except for 15 wt. % TiO_2_. Elongations of nanocomposites with 1–2% TiO_2_ are about 19.1% and 24% higher than the pristine PLA.	[142]
Poly(lactic acid) (PLA)–TiO_2_	*Matrix:* poly(lactic acid) (PLA) *Filler:* TiO_2_ (1, 3, 5, 10 wt.%) *Coupling agent:* c-methacryloxy propyltrimethoxy-silane)	*Obtaining:* in situ polymerization, *Characterization:* DSC, TGA, XDR, SEM, thermal and mechanical properties	The tensile strength, elongation at break, and Young’s modulus of PLA–TiO_2_ (3 wt.%) composites are improved to a certain degree compared with those of pristine PLA.	[143]
**Thermosetting matrix**				
epoxy–TiO_2_ nanocomposites	*Matrix:* mixture (resin + hardener); *Filler:* TiO_2_ (0.5, 1, 2, 3, 4, 5, 8 and 10% vol.);	*Obtaining:* mixing of resin + hardener and filler; *Characterization:* tensile test, dynamic mechanical analysis;	The incorporation of TiO_2_ nanoparticles into the epoxy resin improved flexural stiffness, flexural strength, and fracture toughness of the polymer.	[144]
epoxy–TiO_2_ nanocomposites	*Matrix:* epoxy resin *Filler:* TiO_2_ (5–40 nm, 0.5–2 wt.%);	*Obtaining:* mixing of matrix with filler; *Characterization:* thermal properties, mechanical properties, morphology, viscoelastic properties.	TiO_2_ composites with dimensions between 5–10 nm showed better properties than those with larger dimensions (20–50 nm).	[145]
epoxy–TiO_2_ nanocomposites	*Matrix:* mixture (resin+hardener); *Filler:* TiO_2_ (1, 3, 5, 10 wt.%) *Coupling agent:* methyl isobutyl-ketone; dodecylbenzene-sulfonic acid	*Obtaining:* mixing of matrix, filler and coupling agent; *Characterization:* FTIR, SEM, XRD, TGA, mechanical tests	The mechanical properties of materials are found to improve with TiO_2_, but degrade if the nano-TiO_2_ exceeds 3%.	[146]
epoxy–TiO_2_ nanocomposites	*Matrix:* epoxy resin (DER 331TM) *Filler:* TiO_2_ (220 nm, 50 nm and 17 nm crystal diameter); *Coupling agent:* isophorone diamine (IPDA) + salicylic acid.	*Obtaining:* mixing of matrix, filler and coupling agent; *Characterization:* mechanical test, XPS, SEM	The highest tensile stress values were found at 3 wt. % TiO_2_ (17 nm and 50 nm) and 5 wt. % TiO_2_ (220 nm). The maximum flexural properties were found at a lower TiO_2_ fraction of 1 wt.% only.	[147]
epoxy–TiO_2_ micro and nanocomposites	*Matrix:* epoxy resin:curing agent = 2:1 (wt. %) *Filler:* TiO_2_ (0.2 μm; 1, 5, 10, 15 wt. %); TiO_2_ (21 nm; 0.5, 1, 3 wt. %).	*Obtaining:* mixing with an electrical stirrer, *Characterization:* tensile test, tensile creep-recovery test, tensile stress relaxation tests, SEM.	TiO_2_ nanocomposites have better strength properties than TiO_2_ microcomposites due to the size of the particle.	[148]
vinyl ester resins–TiO_2_ nanocomposites	*Matrix:* vinyl ester:styrene monomers (55:45 wt. %) *Filler:* TiO_2_ (21 nm; 50 m^2^/g; 1, 2.5, and 5 wt. %). *Coupling agent:* polymeric coupling BYK-C 8000	*Obtaining:* shear mixing and ultrasonication; *Characterization:* tensile test, flexural test, impact test, SEM	For nanocomposite with 0–2.5 wt. % TiO_2_, the tensile strength exhibits increasing tendency, while loading more than 2.5 wt. % leads to its decline.	[149]
epoxy resin–polyurethane (EP-PU)–TiO_2_	*Matrix:* EP-PU epoxy resin; *Filler:* TiO_2_ (0.42 g/cm^3^; 25 nm) *Coupling agent:* isopropyl tri(dioctylpyrophosphate) titanate (TCA201)	*Obtaining:* mixing EP–PU and TCA201–TiO_2_ *Characterization:* FT-IR spectroscopy, SEM analysis, TGA analysis, mechanical properties, dielectric constant	The shear strength reached the maximum value (27.14 MPa) for EP–PU/TiO_2_ (3 wt. %) and its thermal decomposition temperature increase by 17.48 º C more than that of EP–PU matrix. The dielectric constant and dielectric loss showed 4.27 and 0.02, respectively.	[85]
**Elastomeric matrix**				
TiO_2_–natural rubber composites	*Matrix:* natural rubber (NR) *Filler:* TiO_2_ (KEMOX RC 800 PG) and the surface-modified nanosilica	*Obtaining:* hydraulic press under a pressure *Characterization:* stress relaxation measurements, SEM, AFM, effect of strain level, effect of ageing	The rate of stress relaxation was higher for silica-filled NR than TiO_2_-filled NR. This is due to the high degree of agglomeration in silica compared to TiO_2_. The relaxation rate increased with increasing TiO_2_ loading.	[150]
TiO_2_–natural rubber composite	*Matrix*: natural rubber stabilised with ammonia; *Filler*: TiO_2_ dispersion (2, 4 and 6 pphr)	*Obtaining:* TiO_2_ dispersions added in matrix; *Characterisation:* tesnsile test	The results showed improvement in both elongations at break and tensile strength data at low filler concentration (2 phr).	[151]
TiO_2_–natural rubber composites	*Matrix*: natural rubber latex centrifuged with ammonia; *Filler:* TiO_2_ (3 mm;.13 g/mL); TiO_2_ (15–40 nm; 4.26 g/mL); *aditives:* zinc oxide, stearic acid, N-cyclohexyl-benzothiazyl-sulphenamide, N2′-propyl-N-phenylenediamine, and S	*Obtaining:* TiO_2_ dispersion was immersed in natural rubber latex. *Characterization:* tensile test, SEM, TEM, XRD	The tensile strength of nano-sized TiO_2_-filled natural rubber composites (23.04 MPa) is superior to micro-sized TiO_2_-filled natural rubber composites (19.62 MPa) (for 6 phr of micro- and nano-s)	[152]
TiO_2_–natural rubber composites	*Matrix*: natural rubber; *Filler:* TiO_2_-15, 25, 45, 85 wt. % *aditives:* stearic acid, sulfur powder and zinc oxide;	*Obtaining:* compression molding; *Characterization:* mechanical properties; dynamic mechanical properties; thermal stability	TiO_2_ as filler allows obtaining materials with improved mechanical properties and thermal stability compared to the pristine natural rubber vulcanizates.	[153]
TiO_2_–chlorobutyl rubber composites	*Matrix:* chlorobutyl rubber (CBK 150) with 1.2% Cl; *Filler:* TiO_2_ (10–30 phr.) *Additives:* stearic acid, zinc oxide, sulfur, and zinc	*Obtaining:* mixing in a two-roll mill *Characterization:* mechanical properties, morphology (SEM, AFM), thermophysical measurements, diffusion experiments	The tensile strength of the composites increases by 250% when the filler loading goes to 40 phr (tensile modulus the same).	[133]
Acrylonitrile–Butadiene–Styrene–TiO_2_ nanocomposites	*Matrix*: acrylonitrile butadiene styrene (ABS) *Fillers:* TiO_2_ (25–50 nm; 0.5, 2.5, 5 and 10 wt. %) and ATO (size < 50 nm)	*Obtaining:* mechanical homogeniser. *Characterization:* SEM, AFM and Raman analysis, thermal properties, tensile test, flexural tests, micro-hardness tests.	The tensile strength of ABS/TiO_2_ and ABS/ATO nanocomposites increased by 7% at the 2.5 wt. % TiO_2_ filler, respectively, by 9.2% at 0.50 wt. % ATO filler. The modulus of elasticity increases up to 5 TiO_2_ wt. % and then decreases.	[31]

When designing new polymer–TiO_2_ nanoparticle composites, the following aspects should be considered:

− Nature of filler and polymer matrix;
− Amount of filler;
− The distribution of filler, this should not form agglomerates in the samples;
− Concentration of coupling agent for modifying of filler surface;
− The method of obtaining, which is an essential factor.

The impact resistance of polymer matrices with TiO_2_ filled is of particular interest to researchers, as long as it represents the weak point of most composite materials. Hardening of thermoplastics by modification with elastomers could be a new way to solve this problem. It is recommended to study new cheaper and more efficient polymer matrices to produce composites with predetermined properties. In this case, we recommend using of polymeric waste as a matrix for obtaining nanocomposites with TiO_2_ filler.

Polymer nanocomposites give a new way to overcome the limitations of pure polymers or their traditional composites. Nowadays, polymer nanocomposites with TiO_2_ filled represent an area of interest for many researchers. This article contains information on the nature of the polymer matrix (thermoplastic, thermosetting, elastomeric) and the type of TiO_2_ filler, processing methods, possible surface modifications of the filler and how they influence the mechanical properties of nanocomposites, thus completing the areas of knowledge for many researchers.

### 4.3. Advantages, Limits and Applications

**Figure 5:**
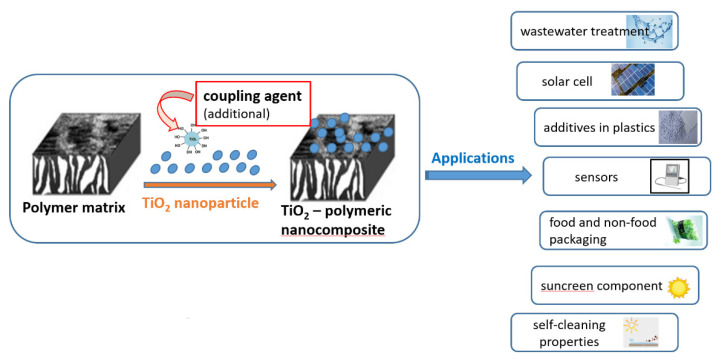
Applications of the TiO_2_ nanocomposites.

Polymeric materials can be used as matrices in TiO_2_ nanocomposites due to their good thermal stability, environmental resistance (durability), and electrical, chemical and mechanical properties. However, it is well known that some polymers (e.g., epoxy resin, polyamides) are highly brittle. This disadvantage limits the application of these polymers in products that require high impact and fracture strength. TiO_2_ filler added in the polymer matrix improves the mechanical performance of the polymeric nanocomposites over conventional polymer composites, as shown in Table 4. Finally, typical existing and potential applications are shown in Figure 5.

## 5. Conclusions

An essential characteristic of polymers is modifying their inherent physical properties by adding fillers, while retaining their characteristic processing ease. By adding inorganic fillers into the polymers matrix, composite materials become stronger, stiffer, electronically conductive, magnetically permeable, flame retardant, more challenging, and more wear-resistant.

After reviewing part of the existing literature on polymeric composites with TiO_2_ fillers, it is found that the interfacial connection between the filler and polymer matrix is an important element for determining the mechanical properties of the composite.

The addition of TiO_2_ nanoparticles into the polymeric matrix demonstrates their ability to significantly improve important mechanical properties (tensile modulus, tensile strength, toughness and fracture toughness, fracture energies, flexural modulus, flexural strength, elongation at break, fatigue crack propagation resistance, abrasion, pull-off strength, and fracture surface properties), even at low filler contents.

From the literature, one can conclude that the mechanical properties of the composites with the polymer matrix depend on the particle size, and particle–matrix interface adhesion and loading (type, quantity, filler distribution and orientation, and void content). Along with those properties, the interfacial bonds and the interphase load mechanisms also play an essential role.

Studies performed on polymeric matrix nanocomposites filled with TiO_2_ nanoparticles were performed to verify the influence of several variables (shape, size, % loading, surface change, etc.) and also to propose various areas of applicability of these nanocomposites.

## Figures and Tables

**Table 1:** Surface modification of TiO_2_ nanoparticles.

Modification Agent of TiO_2_ Surface	Chemical Structure	Polymer–TiO_2_ Nanocomposite	Ref
3-(trimethoxysilyl)propyl methacrylate, KH–570	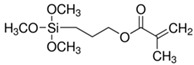	silicone rubber–TiO_2_ nanocomposite	[72]
fluoro silane	H_3_Si–F	HDPE–TiO_2_ nanocomposite	[73]
glycidyl methacrylate	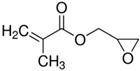	methyl methacrylate–butyl acrylate/dimethylaminoethyl methacrylate–butyl acrylate–acrylic acid–TiO_2_ nanoparticles	[74]
bis-(3-triethoxysilylpropyl) tetrasulfide (TESPT)	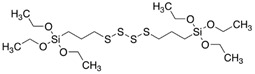	rubber–TiO_2_ nanocomposite	[75]
3-amino propyl trimethoxy silane	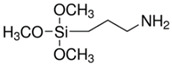	PA11–TiO_2_ nanocomposite;PU-TiO_2_ composites;	[76,77]
3-amino propyl triethoxy silane	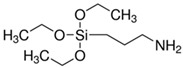	nylon 6/TiO_2_ composites;PS–TiO_2_ microcompositespolyurethane–TiO_2_ composites;polyamide–TiO_2_ nanocomposites	[45,77,78]
hexadecyl trimethoxy silane	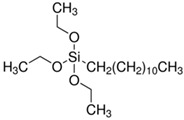	PE–TiO_2_ nanocomposite	[79]
vinyl trimethoxy silane (VTMS)	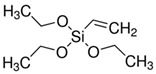	LDPE–TiO_2_ nanocomposite	[80]
6-palmitate ascorbic acid	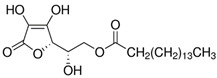	PMMA–TiO_2_ nanocomposite	[81]
3-methacryloxy propyl trimethoxy silane	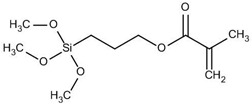	PMMA–TiO_2_ nanocomposite;acrylonitrile–styrene-acrylate terpolymer–TiO_2_ composite;PS-b-PMMA–TiO_2_ nanocomposite	[82,83]
cetyl trimethylammonium chloride (TMAC) amphiphilics	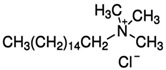	PS-b-PMMA–TiO_2_ nanocomposite	[84]
isopropyl tri(dioctylpyrophosphate) titanate (TCA201)	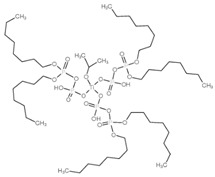	EP-PU/TiO_2_ composite	[85]
3-isocyanato propyl trimethoxy silane	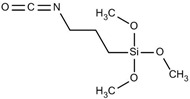	polymer–TiO_2_	[86]

**Table 2:** Some properties of TiO_2_ and applications.

Application	Properties
Photocatalysis	Particularly in anatase from under ultraviolet light
Self-cleaning and anti-fogging glass	Spiked with nitrogen ions or droplet with metal oxides under UV–visible light
Hydrolysis catalyst	Super hydrophilicity, deodorizing, sterilizing, anti-fouling; chemical resistance
Dye-sensitized solar cells	Strong oxidative potential for develop OH radicals
Pigments, opacifiers, cosmetic, UV absorber	Brightness, high reflective index, high reflective optical, perfect white, opacity, nontoxic to human life

**Table 4:** Advantages of polymer nanocomposites over conventional polymer composites.

Polymer Nanocomposites	Conventional Polymer Composites
✓ Fillers separation are in nm, and properties will be affected by size effects of nanofiller; ✓ Small amounts of TiO_2_ filler are enough (less than 10%) to achieve desired properties; ✓ Properties are obtained without sacrificing the inherent properties of the polymer or adding excessive weight; ✓ Improvements in properties even in low amount is due to nanosized of TiO_2_ filler and interphase region; ✓ Using nanosized particles can reduce the likelihood of finding defects, such as grain boundaries, voids, dislocations, and imperfections.	✓ Fillers are separated in μm, and there is not that much of size effect; ✓ High concentrations should be needed as compared to nanofiller case; ✓ Fillers can unfavorably impact other benefits of polymers, such as appearance, ductility and toughness; ✓ There is not that much improvement in properties even for a large amount of fillers; ✓ It is difficult even observed in conventional polymer composites.

## Data Availability

The data presented in this study are available on request from the corresponding author.

## References

[1] Mittal V. (2010). Optimization of Polymer Nanocomposite Properties.

[2] Schadler L.S., Brinson L.C., Sawyer W.G. (2007). Polymer nanocomposites: A small part of the story. JOM.

[3] Young R., Kinloch I.A., Gong L., Novoselov K. (2012). The mechanics of graphene nanocomposites: A review. Compos. Sci. Technol..

[4] Oliviera M., Machado A., Wang X. (2013). Preparation of Polymer-Based Nanocomposites by Different Routes. Nanocomposites: Synthesis, Characterization and Application.

[5] Kumar R. (2014). Polymer-Matrix Composites Types, Applications & Performance.

[6] Seferis J. (2013). Role of the Polymeric Matrix in the Processing and Structural Properties of Composite Materials.

[7] Venditti I., D’Amato R., Russo M.V., Falconieri M. (2007). Synthesis of conjugated polymeric nanobeads for photonic bandgap materials. Sens. Actuators B Chem..

[8] Soares I.L., Chimanowsky J.P., Luetkmeyer L., Da Silva E.O., Souza D.D.H.S., Tavares M.I.B. (2015). Evaluation of the Influence of Modified TiO_2_ Particles on Polypropylene Composites. J. Nanosci. Nanotechnol..

[9] Monteiro M., Neto R.C., Santos I.C.S., Da Silva E.O., Tavares M.I.B. (2012). Inorganic-organic hybrids based on poly (ε-Caprolactone) and silica oxide and characterization by relaxometry applying low-field NMR. Mater. Res..

[10] Rubab Z., Afzal A., Siddiqi H.M., Saeed S. (2014). Preparation, Characterization, and Enhanced Thermal and Mechanical Properties of Epoxy-Titania Composites. Sci. World J..

[11] Kierys A., Zaleski R., Buda W., Pikus S., Dziadosz M., Goworek J. (2012). Nanostructured polymer–titanium composites and titanium oxide through polymer swelling in titania precursor. Colloid Polym. Sci..

[12] Suzuki N., Kiba S., Kamachi Y., Miyamoto N., Yamauchi Y. (2012). Unusual reinforcement of silicone rubber compounds containing mesoporous silica particles as inorganic fillers. Phys. Chem. Chem. Phys..

[13] Da Silva P.S.R.C., Tavares M.I.B. (2015). Solvent Effect on the Morphology of Lamellar Nanocomposites Based on HIPS. Mater. Res..

[14] Cunha A.D.P.C.B., Tavares M.I.B., Silva E.O., Zaioncz S. (2015). The Effect of Montmorillonite Clay on the Crystallinity of Poly(vinyl alcohol) Nanocomposites Obtained by Solution Intercalation and In Situ Polymerization. J. Nanosci. Nanotechnol..

[15] Motaung T., Luyt A., Saladino M., Caponetti E. (2013). Study of morphology, mechanical properties, and thermal degradation of polycarbonate-titania nanocomposites as function of titania crystalline phase and content. Polym. Compos..

[16] Liaw W.-C., Cheng Y.-L., Liao Y.-S., Chen C.-S., Lai S.-M. (2011). Complementary functionality of SiO_2_ and TiO_2_ in polyimide/silica–titania ternary hybrid nanocomposites. Polym. J..

[17] Shishkovsky I.V., Scherbakov V.I. (2021). Additive manufacturing of polymer composites with nano-titania inclusions. Laser Phys. Lett..

[18] Sanes J., Sánchez C., Pamies R., Avilés M.-D., Bermúdez M.-D. (2020). Extrusion of Polymer Nanocomposites with Graphene and Graphene Derivative Nanofillers: An Overview of Recent Developments. Materials.

[19] Müller C.M., Laurindo J.B., Yamashita F. (2012). Composites of thermoplastic starch and nanoclays produced by extrusion and thermopressing. Carbohydr. Polym..

[20] Kickelbick G. (2007). Hybrid Materials: Synthesis, Characterization, and Applications.

[21] Almeida A.D.S., Tavares M.I.B., Da Silva E.O., Neto R.P.C., Moreira L.A. (2012). Development of hybrid nanocomposites based on PLLA and low-field NMR characterization. Polym. Test..

[22] Cheraghian G. (2016). Effect of nano titanium dioxide on heavy oil recovery during polymer flooding. Pet. Sci. Technol..

[23] Vidakis N., Petousis M., Maniadi A., Koudoumas E., Liebscher M., Tzounis L. (2020). Mechanical Properties of 3D-Printed Acrylonitrile–Butadiene–Styrene TiO_2_ and ATO Nanocomposites. Polymers.

[24] Vidakis N., Maniadi A., Petousis M., Vamvakaki M., Kenanakis G., Koudoumas E. (2020). Mechanical and Electrical Properties Investigation of 3D-Printed Acrylonitrile–Butadiene–Styrene Graphene and Carbon Nanocomposites. J. Mater. Eng. Perform..

[25] Nasu A., Otsubo Y. (2007). Rheology and UV-protecting properties of complex suspensions of titanium dioxides and zinc oxides. J. Colloid Interface Sci..

[26] Uddin M., Mondal D.P., Morris C., Lopez T., Diebold U., Gonzalez R.D. (2011). An in vitro controlled release study of valproic acid encapsulated in a titania ceramic matrix. Appl. Surf. Sci..

[27] Fujihara K., Kumar A., Jose R., Ramakrishna S., Uchida S. (2007). Spray deposition of electrospun TiO_2_ nanorods for dye-sensitized solar cell. Nanotechnology.

[28] Andronic L., Enesca A. (2020). Black TiO_2_ Synthesis by Chemical Reduction Methods for Photocatalysis Applications. Front. Chem..

[29] Saccà A., Carbone A., Gatto I., Pedicini R., Freni A., Patti A., Passalacqua E. (2016). Composites Nafion-titania membranes for Polymer Electrolyte Fuel Cell (PEFC) applications at low relative humidity levels: Chemical physical properties and electrochemical performance. Polym. Test..

[30] Fiorati A., Bellingeri A., Punta C., Corsi I., Venditti I. (2020). Silver Nanoparticles for Water Pollution Monitoring and Treatments: Ecosafety Challenge and Cellulose-Based Hybrids Solution. Polymers.

[31] Pantalei S., Zampetti E., Macagnano A., Bearzotti A., Venditti I., Russo M. (2007). Enhanced Sensory Properties of a Multichannel Quartz Crystal Microbalance Coated with Polymeric Nanobeads. Sensors.

[32] Fratoddi I., Cartoni A., Venditti I., Catone D., O’Keeffe P., Paladini A., Toschi F., Turchini S., Sciubba F., Testa G. (2018). Gold nanoparticles functionalized by rhodamine B isothiocyanate: A new tool to control plasmonic effects. J. Colloid Interface Sci..

[33] Sharpe L.H., Akovali G. (1993). The Interfacial Interactions în Polymeric Composites.

[34] Jesson D., Watts J.F. (2012). The Interface and Interphase in Polymer Matrix Composites: Effect on Mechanical Properties and Methods for Identification. Polym. Rev..

[35] Wang M., Wang Z., Li N., Liao J., Zhao S., Wang J., Wang S. (2015). Relationship between polymer–filler interfaces in separation layers and gas transport properties of mixed matrix composite membranes. J. Membr. Sci..

[36] Drzal L.T., Rich M.J., Lloyd P.F. (1983). Adhesion of Graphite Fibers to Epoxy Matrices: I. The Role of Fiber Surface Treatment. J. Adhes..

[37] Berlin A.A., Volfson S.t.A., Enikilopian N.S., Negmatov S.S. (1985). Principles of Polymer Composites.

[38] Cassidy P.E., Yager B.J., Skeist I. (1972). Coupling Agents As Adhesion Promoters în Reviews în Polymer Technology.

[39] Kim J.-K., Mai Y.-W. (1998). Engineered Interfaces in Fiber Reinforced Composites.

[40] Fourche G. (1995). An overview of the basic aspects of polymer adhesion. Part I: Fundamentals. Polym. Eng. Sci..

[41] Morris H.R., Turner J.F., Munro B., Ryntz R.A., Treado P.J. (1999). Chemical Imaging of Thermoplastic Olefin (TPO) Surface Architecture. Langmuir.

[42] Del Rio F.W., De Boer M., Knapp J.A., Reedy E.D., Clews P.J., Dunn M. (2005). The role of van der Waals forces in adhesion of micromachined surfaces. Nat. Mater..

[43] Lipatov Y. (1995). Polymer Reinforcement.

[44] Qin R.-Y., Schreiber H. (1999). Adhesion at partially restructured polymer surfaces. Colloids Surf. A Physicochem. Eng. Asp..

[45] Selvin T.P., Kuruvilla J., Sabu T. (2004). Mechanical properties of titanium dioxide-filled polystyrene microcomposites. Mater. Lett..

[46] De Armitt C., Rothon R. (2002). Fillers and surface treatment. Plast. Addit. Compd..

[47] Fronza B.M., Lewis S., Shah P.K., Barros M.D., Giannini M., Stansbury J.W. (2019). Modification of filler surface treatment of composite resins using alternative silanes and functional nanogels. Dent. Mater..

[48] Mozetič M. (2019). Surface Modification to Improve Properties of Materials. Materials.

[49] Chaudhari S., Shaikh T., Pandey P. (2013). A Review on Polymer TiO_2_ Nanocomposites. Int. J. Eng. Res. Appl..

[50] Dalod A.R.M., Henriksen L., Grande T., Einarsrud M.-A. (2017). Functionalized TiO_2_ nanoparticles by single-step hydrothermal synthesis: The role of the silane coupling agents. Beilstein J. Nanotechnol..

[51] Chen X., Mao S.S. (2006). Synthesis of Titanium Dioxide (TiO_2_) Nanomaterials. J. Nanosci. Nanotechnol..

[52] Tao P., Li Y., Rungta A., Viswanath A., Gao J., Benicewicz B., Siegel R.W., Schadler L.S. (2011). TiO_2_ nanocomposites with high refractive index and transparency. J. Mater. Chem..

[53] Díez-Pascual A.M., Díez-Vicente A.L. (2015). Nano-TiO_2_ Reinforced PEEK/PEI Blends as Biomaterials for Load-Bearing Implant Applications. ACS Appl. Mater. Interfaces.

[54] Byranvand M.M., Kharat A.N., Fatholahi L., Beiranvand Z.M. (2013). A review on synthesis of nano-TiO_2_ via different methods. JNS.

[55] Wang Y., He Y., Lai Q., Fan M. (2014). Review of the progress in preparing nano TiO_2_: An important environmental engineering material. J. Environ. Sci..

[56] Hanemann T., Szabó D.V. (2010). Polymer-Nanoparticle Composites: From Synthesis to Modern Applications. Materials.

[57] Laine R.M., Choi J., Lee I. (2001). Organic–Inorganic Nanocomposites with Completely Defined Interfacial Interactions. Adv. Mater..

[58] Li H., Zhang Z., Ma X., Hu M., Wang X., Fan P. (2007). Synthesis and characterization of epoxy resin modified with nano-SiO2 and γ-glycidoxypropyltrimethoxy silane. Surf. Coat. Technol..

[59] Xu X., Li B., Lu H., Zhang Z., Wang H. (2007). The interface structure of nano-SiO_2_/PA66 composites and its influence on material’s mechanical and thermal properties. Appl. Surf. Sci..

[60] Li X., Cao Z., Zhang Z., Dang H. (2006). Surface-modification in situ of nano-SiO_2_ and its structure and tribological properties. Appl. Surf. Sci..

[61] Iijima M., Sato N., Lenggoro I.W., Kamiya H. (2009). Surface modification of BaTiO_3_ particles by silane coupling agents in different solvents and their effect on dielectric properties of BaTiO3/epoxy composites. Colloids Surf. A Physicochem. Eng. Asp..

[62] Balas F., Kokubo T., Kawashita M., Nakamura T. (2007). Surface modification of organic polymers with bioactive titanium oxide without the aid of a silane-coupling agent. J. Mater. Sci. Mater. Electron..

[63] Zhang Y., Chen H., Wen Y., Yuan Y., Wu W., Liu C. (2014). Tunable wettability of monodisperse core-shell nano-SiO_2_ modified with poly(methylhydrosiloxane) and allyl-poly(ethylene glycol). Colloids Surf. A Physicochem. Eng. Asp..

[64] Plueddemann E.P. (1991). Silane Coupling Agents.

[65] Yosomiya R., Morimoto K., Nakajima A., Ikada Y., Suzuki T., Dharan C.K.H. (1991). Adhesion and Bonding in Composites. J. Eng. Ind..

[66] Konakanchi A., Alla R.M., Guduri V. (2017). Silane Coupling Agents—Benevolent Binders in Composites. Trends Biomater. Artif. Organs.

[67] Shokoohi S., Arefazar A., Khosrokhavar R. (2008). Silane Coupling Agents in Polymer-based Reinforced Composites: A Review. J. Reinf. Plast. Compos..

[68] Zhang Y., Fang F., Wang C., Wang L., Wang X., Chu X., Li J., Fang X., Wei Z., Wang X. (2014). Hydrophobic modification of ZnO nanostructures surface using silane coupling agent. Polym. Compos..

[69] Mallakpour S., Madani M. (2014). The effect of the coupling agents KH550 and KH570 on the nanostructure and interfacial interaction of zinc oxide/chiral poly(amide–imide) nanocomposites containing l-leucine amino acid moieties. J. Mater. Sci..

[70] Liu Y.-L., Su Y.-H., Lai J.-Y. (2004). In situ crosslinking of chitosan and formation of chitosan–silica hybrid membranes with using γ-glycidoxypropyltrimethoxysilane as a crosslinking agent. Polymer.

[71] Lu Y., Zhou S., Wu L. (2012). De-Agglomeration and Dispersion Behavior of TiO_2_ Nanoparticles in Organic Media Using 3-Methacryloxypropyltrimethoxysilane as a Surface Modifier. J. Dispers. Sci. Technol..

[72] Dang Z.-M., Xia Y.-J., Zha J.-W., Yuan J., Bai J. (2011). Preparation and dielectric properties of surface modified TiO_2_ /silicone rubber nanocomposites. Mater. Lett..

[73] Xu Q.F., Liu Y., Lin F.-J., Mondal B., Lyons A.M. (2013). Superhydrophobic TiO_2_ –Polymer Nanocomposite Surface with UV-Induced Reversible Wettability and Self-Cleaning Properties. ACS Appl. Mater. Interfaces.

[74] Rahim-Abadi M.M., Mahdavian A.R., Gharieh A., Salehi-Mobarakeh H. (2015). Chemical modification of TiO_2_ nanoparticles as an effective way for encapsulation in polyacrylic shell via emulsion polymerization. Prog. Org. Coat..

[75] Toh-Ae P., Junhasavasdikul B., Lopattananon N., Sahakaro K. (2013). Surface Modification of TiO_2_ Nanoparticles by Grafting with Silane Coupling Agent. Adv. Mater. Res..

[76] Ambrósio J.D., Balarim C.V.M., De Carvalho G.B. (2014). Preparation, characterization, and mechanical/tribological properties of polyamide 11/Titanium dioxide nanocomposites. Polym. Compos..

[77] Sabzi M., Mirabedini S.M., Zohuriaan-Mehr M.J., Atai M. (2009). Surface modification of TiO_2_ nano-particles with silane coupling agent and investigation of its effect on the properties of polyurethane composite coating. Prog. Org. Coat..

[78] Rusu G., Rusu E. (2011). Nylon 6/TiO2Composites by in situ Anionic Ring-Opening Polymerization of ϵ-Caprolactam: Synthesis, Characterization, and Properties. Int. J. Polym. Anal. Charact..

[79] Zapata P.A., Palza H., Delgado K., Rabagliati F.M. (2012). Novel antimicrobial polyethylene composites prepared by metallocenic in situ polymerization with TiO_2_-based nanoparticles. J. Polym. Sci. Part A Polym. Chem..

[80] Nguyen V.G., Thai H., Mai D.H., Tran H.T., Tran D.L., Vu M.T. (2013). Effect of titanium dioxide on the properties of polyethylene/ TiO_2_ nanocomposites. Compos. Part B Eng..

[81] Dzžunuzović E., Marinović-Cincović M., Vuković J., Jeremić K., Nedeljkovic J. (2009). Thermal properties of PMMA/ TiO_2_ nanocomposites prepared byin-situbulk polymerization. Polym. Compos..

[82] Yuvaraj H., Kim W.S., Kim J.T., Kang I.P., Gal Y.-S., Kim S.W., Lim K.T. (2009). Synthesis of Poly(methyl methacrylate) Encapsulated TiO_2_ Nanocomposite Particles in Supercritical CO_2_. Mol. Cryst. Liq. Cryst..

[83] Qi Y., Xiang B., Tan W., Zhang J. (2017). Hydrophobic surface modification of TiO_2_ nanoparticles for production of acrylonitrile-styrene-acrylate terpolymer/ TiO_2_ composited cool materials. Appl. Surf. Sci..

[84] Weng C.-C., Wei K.-H. (2003). Selective Distribution of Surface-Modified TiO_2_ Nanoparticles in Polystyrene-b-poly (Methyl Methacrylate) Diblock Copolymer. Chem. Mater..

[85] Yufei C., Zhichao L., Junyan T., Qingyu Z., Yang H. (2015). Characteristics and Properties of TiO_2_ /EP-PU Composite. J. Nanomater..

[86] Zhao J., Milanova M., Warmoeskerken M.M., Dutschk V. (2012). Surface modification of TiO_2_ nanoparticles with silane coupling agents. Colloids Surf. A Physicochem. Eng. Asp..

[87] Wanag A., Sienkiewicz A., Rokicka-Konieczna P., Kusiak-Nejman E., Morawski A.W. (2020). Influence of modification of titanium dioxide by silane coupling agents on the photocatalytic activity and stability. J. Environ. Chem. Eng..

[88] Mallakpour S., Barati A. (2011). Efficient preparation of hybrid nanocomposite coatings based on poly(vinyl alcohol) and silane coupling agent modified TiO2 nanoparticles. Prog. Org. Coat..

[89] Shakeri A., Yip D., Badv M., Imani S.M., Sanjari M., Didar T.F. (2018). Self-Cleaning Ceramic Tiles Produced via Stable Coating of TiO2 Nanoparticles. Materials.

[90] Klaysri R., Tubchareon T., Praserthdam P. (2017). One-step synthesis of amine-functionalized TiO2 surface for photocatalytic decolorization under visible light irradiation. J. Ind. Eng. Chem..

[91] Chen Q., Yakovlev N.L. (2010). Adsorption and interaction of organosilanes on TiO2 nanoparticles. Appl. Surf. Sci..

[92] Dinari M., Haghighi A. (2017). Surface modification of TiO_2_ nanoparticle by three dimensional silane coupling agent and preparation of polyamide/modified-TiO_2_ nanocomposites for removal of Cr (VI) from aqueous solutions. Prog. Org. Coat..

[93] Caris C., Kuijpers R., Van Herk A.M., German A.L. (1990). Kinetics of (CO)polymerizations at the surface of inorganic submicron particles in emulsion-like systems. Makromol. Chem. Macromol. Symp..

[94] Erdem B., Sudol E.D., Dimonie V.L., El-Aasser M.S. (2000). Encapsulation of inorganic particles via miniemulsion polymerization. II. Preparation and characterization of styrene miniemulsion droplets containing TiO_2_ particles. J. Polym. Sci. Part A Polym. Chem..

[95] Rong M.Z., Zhang M.Q., Wang H.B., Zeng H.M. (2002). Surface modification of magnetic metal nanoparticles through irradiation graft polymerization. Appl. Surf. Sci..

[96] Yang M., Dan Y. (2005). Preparation and characterization of poly(methyl methacrylate)/titanium oxide composite particles. Colloid Polym. Sci..

[97] Milanesi F., Cappelletti G., Annunziata R., Bianchi C.L., Meroni D., Ardizzone S. (2010). Siloxane− TiO_2_ Hybrid Nanocomposites. The Structure of the Hydrophobic Layer. J. Phys. Chem. C.

[98] Xiang B., Jiang G., Zhang J. (2015). Surface modification of TiO_2_ nanoparticles with silane coupling agent for nanocomposite with poly(butyl acrylate). Plast. Rubber Compos..

[99] Wang C., Mao H., Wang C., Fu S. (2011). Dispersibility and Hydrophobicity Analysis of Titanium Dioxide Nanoparticles Grafted with Silane Coupling Agent. Ind. Eng. Chem. Res..

[100] Godnjavec J., Znoj B., Vince J., Steinbucher M., Žnidaršič A., Venturini P. (2012). Stabilization of rutile TiO_2_ nanoparticles with Glymo in polyacrylic clear coating. Mater. Tehnol..

[101] Yang C., Yang C. (2014). Preparation of TiO_2_ particles and surface silanization modification for electronic ink. J. Mater. Sci. Mater. Electron..

[102] Tangchantra N., Kruenate J., Aumnate C., Sooksomsong T. (2010). The Effect of Surface Modification of TiO_2_ on Mechanical Properties of Polyethylene Composite Film. Adv. Mater. Res..

[103] Fujishima A., Rao T.N., Tryk D.A. (2000). Titanium dioxide photocatalysis. J. Photochem. Photobiol. C Photochem. Rev..

[104] Herrmann J.-M. (1999). Heterogeneous photocatalysis: Fundamentals and applications to the removal of various types of aqueous pollutants. Catal. Today.

[105] Nada A., Barakat M., Hamed H., Mohamed N., Veziroglu T. (2005). Studies on the photocatalytic hydrogen production using suspended modified TiO_2_ photocatalysts. Int. J. Hydrogen Energy.

[106] Andronic L., Enesca A., Cazan C., Visa M. (2014). TiO2–active carbon composites for wastewater photocatalysis. J. Sol.-Gel. Sci. Technol..

[107] Sharma S.K., Vishwas M., Rao K.N., Mohan S., Reddy D.S., Gowda K. (2009). Structural and optical investigations of TiO_2_ films deposited on transparent substrates by sol–gel technique. J. Alloys Compd..

[108] Bayarri B., Giménez J., Curcó D., Esplugas S. (2005). Photocatalytic degradation of 2,4-dichlorophenol by TiO_2_ /UV: Kinetics, actinometries and models. Catal. Today.

[109] Gaya U.I., Abdullah A.H. (2008). Heterogeneous photocatalytic degradation of organic contaminants over titanium dioxide: A review of fundamentals, progress and problems. J. Photochem. Photobiol. C Photochem. Rev..

[110] Shimizu N., Ninomiya K., Ogino C., Rahman M.M. (2010). Potential uses of titanium dioxide in conjunction with ultrasound for improved disinfection. Biochem. Eng. J..

[111] Tuan N.M., Nha N.T., Tuyen N.H. (2009). Low-temperature synthesis of nano-TiO_2_ anatase on nafion membrane for using on DMFC. J. Phys. Conf. Ser..

[112] Armstrong A.R., Armstrong G., Canales-Vazquez J., García R., Bruce P.G. (2005). Lithium-Ion Intercalation into TiO_2_-B Nanowires. Adv. Mater..

[113] Richards B.S., Cotter J.E., Honsberg C.B. (2002). Enhancing the surface passivation of TiO_2_ coated silicon wafers. Appl. Phys. Lett..

[114] Pinto D., Bernardo L., Amaro A., Lopes S. (2015). Mechanical properties of epoxy nanocomposites using titanium dioxide as reinforcement—A review. Constr. Build. Mater..

[115] Sahu M., Satapathy A. Thermal Characteristics of Polypropylene Composites Filled with TiO_2_. Proceedings of the ASEAI 2014.

[116] Peng X., Ding E., Xue F. (2012). In situ synthesis of TiO2/polyethylene terephthalate hybrid nanocomposites at low temperature. Appl. Surf. Sci..

[117] Mourad A.-H.I., Mozumder M.S., Mairpady A., Pervez H., Kannuri U.M. (2017). On the Injection Molding Processing Parameters of HDPE-TiO2 Nanocomposites. Materials.

[118] Zhil’Tsova T., Oliveira M., Ferreira J. (2009). Relative influence of injection molding processing conditions on HDPE acetabular cups dimensional stability. J. Mater. Process. Technol..

[119] Serrano C., Cerrada M., Fernandez-Garcia M., Ressia J., Valles E.M. (2012). Rheological and structural details of biocidal iPP-TiO_2_ nanocomposites. Eur. Polym. J..

[120] Umek P., Huskić M., Škapin A.S., Florjančič U., Zupančič B., Emri I., Arčon D. (2008). Structural and mechanical properties of polystyrene nanocomposites with 1D titanate nanostructures prepared by an extrusion process. Polym. Compos..

[121] Somani P.R., Marimuthu R., Mulik U., Sainkar S., Amalnerkar D. (1999). High piezoresistivity and its origin in conducting polyaniline/TiO2 composites. Synth. Met..

[122] Feng W., Sun E., Fujii A., Wu H., Niihara K., Yoshino K. (2000). Synthesis and Characterization of Photoconducting Polyaniline-TiO2 Nanocomposite. Bull. Chem. Soc. Jpn..

[123] Xia H., Wang Q. (2002). Ultrasonic Irradiation: A Novel Approach To Prepare Conductive Polyaniline/Nanocrystalline Titanium Oxide Composites. Chem. Mater..

[124] Alghamdi M.N. (2016). Titanium Dioxide Reinforced Polypropylene Composites: Preparation and Characterization PP-TiO_2_ Composites. Int. J. Eng. Res. Technol..

[125] Vladuta C., Andronic L., Duta A. (2010). Effect of TiO_2_, nanoparticles on the interface in the PET-rubber composites. J. Nanosci. Nanotechnol..

[126] Mikešová J., Šlouf M., Gohs U., Popelková D., Vacková T., Vu N.H., Kratochvíl J., Zhigunov A. (2014). Nanocomposites of polypropylene/titanate nanotubes: Morphology, nucleation effects of nanoparticles and properties. Polym. Bull..

[127] Sreekumar P., Al-Harthi M.A., De S. (2012). Reinforcement of starch/polyvinyl alcohol blend using nano-titanium dioxide. J. Compos. Mater..

[128] Bora M.Ö., Çoban O., Avcu E., Fidan S., Sınmazçelik T. (2013). The effect of TIO2 filler content on the mechanical, thermal, and tribological properties of TiO_2_ /PPS composites. Polym. Compos..

[129] Saluja P.S., Tiwari J.K., Gupta G. (2017). Preparation and Thermal Behaviour of Polyester Composite Filled with TiO_2_. Int. Res. J. Eng. Technol..

[130] Byrne M.T., McCarthy J.E., Bent M., Blake R., Gun’Ko Y.K., Horvath E., Konya Z., Kukovecz A., Kiricsi I., Coleman J.N. (2007). Chemical functionalisation of titania nanotubes and their utilisation for the fabrication of reinforced polystyrene composites. J. Mater. Chem..

[131] Dong Y., Gui Z., Hu Y., Wu Y., Jiang S. (2012). The influence of titanate nanotube on the improved thermal properties and the smoke suppression in poly(methyl methacrylate). J. Hazard. Mater..

[132] Alexandru M. (2011). On the morphology and potential application of polydimethylsiloxane-silica-titania composites. Express Polym. Lett..

[133] Saritha A., Joseph K., Boudenne A., Thomas S. (2011). Mechanical, thermophysical, and diffusion properties of TiO2-filled chlorobutyl rubber composites. Polym. Compos..

[134] Manap A., Mahalingam S., Vaithylingam R., Abdullah H. (2020). Mechanical, thermal and morphological properties of thermoplastic polyurethane composite reinforced by multi-walled carbon nanotube and titanium dioxide hybrid fillers. Polym. Bull..

[135] Esthappan S.K., Kuttappan S.K., Joseph R. (2012). Thermal and mechanical properties of polypropylene/titanium dioxide nanocomposite fibers. Mater. Des..

[136] Awang M., Mohd W.R.W., Sarifuddin N. (2019). Study the effects of an addition of titanium dioxide (TiO_2_) on the mechanical and thermal properties of polypropylene-rice husk green composites. Mater. Res. Express.

[137] Huang J., Lu X., Zhang N., Yang L., Yan M., Liu H., Zhang G., Qu J. (2013). Study on the properties of nano-TiO_2_ /polybutylene succinate composites prepared by vane extruder. Polym. Compos..

[138] Bragaglia M., Cherubini V., Nanni F. (2020). PEEK-TiO_2_ composites with enhanced UV resistance. Compos. Sci. Technol..

[139] Farhoodi M., Dadashi S., Mousavi S.M.A., Sotudeh-Gharebagh R., Emam-Djomeh Z., Oromiehie A., Hemmati F. (2012). Influence of TiO2 Nanoparticle Filler on the Properties of PET and PLA Nanocomposites. Polym. Korea.

[140] Lu X.-L., Lü X.-Q., Wang J.-Y., Sun Z.-J., Tong Y.-X. (2013). Preparation and shape memory properties of TiO2/PLCL biodegradable polymer nanocomposites. Trans. Nonferrous Met. Soc. China.

[141] Alberton J., Martelli S.M., Fakhouri F.M., Soldi V. (2014). Mechanical and moisture barrier properties of titanium dioxide nanoparticles and halloysite nanotubes reinforced polylactic acid (PLA). IOP Conf. Ser. Mater. Sci. Eng..

[142] Zhang H., Huang J., Yang L., Chen R., Zou W., Lin X., Qu J. (2015). Preparation, characterization and properties of PLA/TiO_2_ nanocomposites based on a novel vane extruder. RSC Adv..

[143] Zhuang W., Liu J., Zhang J.H., Hu B.X., Shen J. (2009). Preparation, characterization, and properties of TiO2/PLA nanocomposites by in situ polymerization. Polym. Compos..

[144] Wetzel B., Rosso P., Haupert F., Friedrich K. (2006). Epoxy nanocomposites—Fracture and toughening mechanisms. Eng. Fract. Mech..

[145] Chatterjee A., Islam M.S. (2008). Fabrication and characterization of TiO_2_–epoxy nanocomposite. Mater. Sci. Eng. A.

[146] Huang K.S., Nien Y.H., Chen J.S., Shieh T.R., Chen J.W. (2006). Synthesis and properties of epoxy/TiO_2_ composite materials. Polym. Compos..

[147] Al-Turaif H.A. (2010). Effect of nano TiO_2_ particle size on mechanical properties of cured epoxy resin. Prog. Org. Coat..

[148] Papanicolaou G., Kontaxis L., Manara A. (2016). Viscoelastic behaviour and modelling of nano and micro TiO_2_ powder-epoxy resin composites. Ciênc. Tecnol. Mater..

[149] Salehian H., Jahromi S.A.J. (2015). Effect of titanium dioxide nanoparticles on mechanical properties of vinyl ester-based nanocomposites. J. Compos. Mater..

[150] Meera A.P., Said S., Grohens Y., Luyt A.S., Thomas S. (2009). Tensile Stress Relaxation Studies of TiO2 and Nanosilica Filled Natural Rubber Composites. Ind. Eng. Chem. Res..

[151] Ochigbo S.S., Luyt A.S. (2011). Mechanical and Morphological Properties of Films Based on Ultrasound Treated Titanium Dioxide Dispersion/Natural Rubber Latex. Int. J. Compos. Mater..

[152] Hayeemasae N., Rathnayake W., Ismail H. (2017). Nano-sized TiO_2_-reinforced natural rubber composites prepared by latex compounding method. J. Vinyl Addit. Technol..

[153] Datta J., Kosiorek P., Włoch M. (2016). Effect of high loading of titanium dioxide particles on the morphology, mechanical and thermo-mechanical properties of the natural rubber-based composites. Iran. Polym. J..

